# Monitoring the progression of atherosclerotic plaque and disruption with contrast-enhancement MR imaging: an experimental study

**DOI:** 10.1186/1532-429X-17-S1-P71

**Published:** 2015-02-03

**Authors:** Lin Yang, Lei Zhao

**Affiliations:** 1 Beijing Anzhen Hospital, Capital Medical University, Beijing, China

## Background

High-resolution CE-MRI is a noninvasive imaging modality to monitor the progression and regression of atherosclerotic plaque and disruption.

The purpose of this study is to monitor the progression of atherosclerotic plaque in rabbit model with serial high-resolution contrast enhancement MRI (CE MRI), and sought to differentiate the morphology and composition between ruptured and non-ruptured plaques before triggering.

## Methods

The animal model was induced in 12 rabbits through endothelial denudation combined with cholesterol-enriched diet; another 4 age- and sex-matched rabbits were enrolled as controls. The plaque disruption was pharmacological triggered by injection of Russell's viper venom in all animals at the end of 3^rd^ month. MR scans were performed at the end of 1^st^ month, 2^nd^ month, pre-trigger and 24 hours post-trigger. Contiguous cross-sectional images of the abdominal aorta were obtained with high resolution multi-slice black blood double inversion-recovery (DIR) T1WI (Pre- and Post-contrast) and T2WI. Two reviewers independently reviewed the imaging datasets and identified the types of plaque, compared with corresponding histopathological sections of the aortic specimens. The percentage of aortic wall area and the percent that thrombosis in the lumen area were determined on different time points MR images by manually tracing the inner and outer vessel wall.

## Results

The average percentage of wall area was increased from 19.11%, 37.34% to 58.19% at the end of 1^st^ month, 2^nd^ month and 3^rd^ month (pre-trigger, P<0.01). Plaque could be detected in 12/12(100%) experimental rabbits and 0/4(0%) in control rabbits with gross anatomy and histological staining, and 9/16 (all in experimental group) rabbits were identified disrupted and intraluminal thrombus post-trigger by CE-MRI. The sensitivity, specificity, and accuracy of MR for detecting thrombus were 91%, 88%, 90%, respectively. However, retrospective assessment of ruptured plaques on the pre-trigger MRI failed to identify any unique attributes relative to areas of the aorta and wall thickness or morphology of plaque that compared with non-ruptured plaques.

## Conclusions

High-resolution CE MRI was referred to monitor the progression and disruption of atherosclerotic plaque. The pre-trigger plaque morphological changes seem to be little value in prediction of experimental disruption and thrombosis of atherosclerotic plaque in rabbit model, although this needs to be further validated.

## Funding

N/A.

